# Factors predicting home storage of medicines in Northern Uganda

**DOI:** 10.1186/1471-2458-14-650

**Published:** 2014-06-26

**Authors:** Moses Ocan, Godfrey S Bbosa, Paul Waako, Jasper Ogwal-Okeng, Celestino Obua

**Affiliations:** 1 Department of Pharmacology & Therapeutics, Makerere University, P.O. Box 7072, Kampala, Uganda; 2 Faculty of Medicine, Gulu University, P.O. Box 166, Gulu, Uganda

**Keywords:** Medicines, Home drug storage, Utilization, Availability, Northern Uganda

## Abstract

**Background:**

Medicines are kept in households Worldwide for first aid, treatment of chronic or acute disease conditions. This promotes inappropriate use of medicines and hence the associated risks. The study explored the factors which predict availability and utilization of medicines in households of Northern Uganda.

**Method:**

A cross sectional survey of 892 households was performed from November-to-December 2012. Five data collectors administered the questionnaires, respondents were requested to bring out any medicines present in their households. Demographic characteristics, drug name, quantity, source, formulation, legibility of drug labels and reasons why the medicines were being kept at home was collected. Data was analyzed using STATA 12.0 at 95% level of significance.

**Results:**

Of the households visited, 35.1% (313/892) had drugs. Paracetamol (11.8%), coartem (11.3%), cotrimoxazole (10%), amoxicillin (9.2%) and metronidazole (8.2%) were the major medicines found. Antibacterial drugs were the most commonly (40.1%) kept type of drugs. The medicines present in households were for on-going treatment (48%); ‘leftover’ (30.5%) and anticipated future use (21.6%). Symptoms of malaria (34.1%) were common in households which had drugs. The medicines kept in homes were mainly from the private sector 60.5% (497/821). The rate of home drug storage was higher 85.3% (267/313) amongst the educated individuals. There was high prevalence 76% (238/313) of self-medication among respondents in households which stored drugs. The average number of medicines in each household was 6 ± 5 with majority (68.1%) having between 1–10 drugs. Previous successful treatment (OR: 1.3; 95% CI: 0.95-1.77), regular income (OR: 1.8; 95% CI: 1.2-2.6) and sex (OR: 0.63; 95% CI: 0.5-0.9) predicted storage of medicines in households in northern Uganda.

**Conclusion:**

Over a third of households in Northern Uganda store medicines with antibacterial agents being the most common. Self-medication is common among individuals in households which keep drugs. Past successful treatment, regular income and sex predict community home drug storage.

## Background

In most households worldwide, medicines are kept for various purposes including emergency use and treatment of chronic or acute illnesses. These medicines are either prescribed by health professionals or obtained over-the-counter in the communities [1]. A study done in Sudan found that about 97.7% of households had at least one medicine in a home medicines cabinet with antimicrobial and analgesic drugs being the common medicines kept in homes [2].

The presence of medicines in households is a risk factor for irrational drug use due mainly to the easy access [2-4]. This exposes patients to adverse drug effects and treatment failures [2]. In most communities of developing countries, there is limited knowledge among the population on the safety of drugs commonly found in homes. The medicines stored in homes are mainly obtained from drug shops, pharmacies and public health facilities [4].

The increasing disease burden especially in developing countries, desire for quick recovery from illness and the acceptance of self-medication among communities influences home storage of drugs [2,4]. Challenges in healthcare delivery such as inadequate access, lack of medical personnel and frequent drug stock outs common in developing countries may influence communities to store drugs in homes [2]. Northern Uganda suffered from two decades of armed conflict which affected the health infrastructure and this could have implications on utilization of healthcare services by the communities.

The challenges of having medicines in homes include poor storage as conditions such as humidity, and temperature are not regulated. This increases the risk of deterioration and expiry of medicines [5]. Due to lack of capacity to detect expired drugs in households; these medicines are in most cases taken by the residents, increasing the risk of adverse effects [3]. Controlling the use of drugs stored at home is a great task especially from unintentional users such as children which increases the risk of accidental poisoning [6]. Presence of medicines at home has also been associated with sharing of drugs which further increase the risk of inappropriate drug use and hence emergence of antimicrobial resistance [6].

Health professionals often focus on giving patients information on medicine use with limited information offered on storage and their disposal [4]. The medicines that inevitably remain after most treatments are disposed in various ways such as throwing in garbage pits and latrines/toilets. This inappropriate disposal of medicines poses danger to the community and the environment [4].

Northern Uganda bore the brunt of more than two decades of armed conflict against the Government of Uganda. Large populations were displaced into internally displaced peoples’ (IDPs) camps. This greatly affected delivery of services in in all sectors but specifically in the health sector. Although peace has now returned to the region with the end of insurgence, and the IDP camps disbanded, the region still lags behind other regions in attracting and retaining health professionals. This is mainly due to inadequate health infrastructures including insufficient medical supplies [7]. The practice of storage of medicines in homes in such environments is more likely as an avenue to improve access to medicines and immediate health care among the population. However, the implication of having medicines in homes has not been fully quantified in most parts of the world especially in developing countries. This study therefore explored factors that predict the storage of medicines in households in Northern Uganda.

## Methods

### Study design

A household survey was carried out between November and December 2012 in communities of Northern Uganda. The study covered four districts including Gulu, Nwoya (Acholi sub-region), Lira, and Dokolo (Lango sub-region). A sample size of 884 households was calculated using a formula for cluster sampling [8]. The following assumptions were considered during the sample size calculation; design effect (2.0), average household size in Northern Uganda (5.0) and 95% level of confidence. In each cluster, households were selected using systematic random sampling with replacement. In each household, data was collected from the household head or any adult member (≥18 years) present at home during the time of data collection.

### Questionnaires and data collection

Data on the presence of medicines in households and their utilization was collected using a structured interviewer administered questionnaire. The questionnaire was pre-tested on twenty households and any questions that was not useful was removed and the useful information that had not been captured by the questionnaire was incorporated by developing appropriate questions. In addition, previous similar studies also provided information that was used in the validation of the study questionnaire [2,4]. The questionnaires were administered by five trained final year diploma pharmacy students from Allied Health Professional Institute, Uganda, and consisted of key questions that sought to establish; i) demographic information regarding sex, age, number of members in the household, marital status, employment status, occupation, income/month, educational background; ii) type of health problem or disease symptoms experienced in the last three months; iii) any medicines presently kept at home; iv) details of the medicines kept at home including the name, formulation, source, prescriber, reason for the medicine being kept in the household, legibility of label and quantity kept; v) length of time taken to reach the source of medicines; vi) awareness of regulations regarding use of medicines; vii) and how any remaining medicines are disposed off after initial treatment.

### Data management and analysis

Data collection tools were checked for completeness at the end of each field day and any inconsistencies resolved by discussing with the data collectors. Double data entry was done in Epi-info 3.1.2 screen created using logic checks. Data was then exported to and analyzed using Stata 12.0 (Stata Corp, College Station, Texas USA). Statistical inferences were made at a 95% level of significance.

Proportions and two-sided chi-square test were performed for prevalence assessments. Multiple logistic regression model was built using backward elimination method and Goodness-of-fit tested using Hosmer-Lemeshow (H-L) method. The model was used to determine the predictors of home storage of drugs in northern Uganda.

### Ethical considerations

The study was approved by Makerere University School of Medicine Research and Ethics Committee (protocol number: REC REF 2012–072) and Uganda National Council of Science and Technology (HS 1267). Permission to collect data in the communities was obtained from local district leadership and village health team members were used as guides through the villages during data collection. The data collection in each household lasted on average between 30–45 minutes.

## Results

### Socio-demographic characteristics

A total of 892 households were visited during the data collection period from November-to-December 2012. The majority of respondents 74.2% (662/892) in the households visited were females. A high proportion 60.6% (541/892) of the respondents reported not to be engaged in any formal employment while 50.6% (451/892) were peasant farmers. Most of the respondents, 72.6% (626/892) reported having no regular income. Majority of the respondents, 82.3% (734/892) had attained a minimum of primary level of education (Table 1).

**Table 1:** Socio-demographic characteristics of respondents

	**Number of respondents (n = 892)**			**P-value Chi-square**
**Characteristic**	**Description**	**Number (n)**	**Proportion (%) who keep drugs at home**	
Sex	Female	662	246 (27.5%)	0.028
	Male	230	67 (7.5%)	4.832
Age	18-26 yr	271	89 (10%)	0.609
	27-35 yr	219	84 (9.3%)	1.828
	36-44 yr	155	56 (6.2%)	
	≥45 yr	247	84 (9.3%)	
Number of members in household	1-4	267	95 (10.6%)	0.964
				0.0735
	5-9	505	177 (19.8%)	
	≥10	120	41 (4.6%)	
Occupation	Peasant farmer	614	204 (22.9%)	0.150
	Business person	72	30 (3.4%)	6.741
	Professional	82	31 (3.5%)	
	No response	124	48 (5.4%)	
Income Ugshs (‘000’)	<10	230	74 (8.3%)	0.190
	11-50	276	106 (11.9%)	6.123
	51-100	129	52 (5.8%)	
	101-250	61	28 (3.1%)	
	251 +	64	20 (2.2%)	
	None response	132	33 (3.7%)	
Level of education	None	158	46 (5.1%)	0.117
	Primary school	477	169 (18.9%)	7.393
	Secondary school	203	72 (8.0%)	
	Tertiary	54	26 (2.9%)	
Time taken to the source of drugs (Minutes)	≤15	333	113 (12.6%)	0.349
				3.289
	16-30	231	75 (8.3%)	
	31-60	186	75 (8.3%)	
	≥61	142	49 (5.5%)	

IQR: Interquartile range: n: sample size; %: Percentage: Ugshs: Ugandan shillings.

### Prevalence of home storage of medicines

Of the households visited, 35.1% (313/892) had stored drugs. Female respondents were more likely to report presence of medicines in their households, 78.6% (246/313; OR: 0.63, 95% CI, 0.45-0.89). The study found that most respondents who earned between 10,000-50,000 Uganda shillings (USD 4–20) kept medicines in their homes 30.9% (276/892) (Table 1).

One in every seven respondents (126/892) reported having regular income of which 47.6% (60/126) kept medicines in their homes (P = 0.007). Respondents who lived between half to one hour distance of travel to the source of medicines were more likely (40.3%) to have medicines kept in their houses (Table 1). Of the respondents who had attained a minimum of primary level of education, 36.4% (267/734) kept medicines in their households.

The medicines kept in households were mainly from the private sector, 60.5% (497/821). The other medicines were obtained from health facilities 38% (312/821), gifts from friends and relatives 0.6%. Solid dosage formulations (tablets/capsules) were the most common, (93.8%) form of medicines kept in households. The other formulations included injections, 3.7%, and ointments, 1.0%.

Antibacterial (40.1%), analgesics (19.6%) and antimalarial (15.6%) drugs were the most common categories of medicines kept in households. Specifically, the following were the major drugs found in most households; paracetamol (11.8%), coartem (11.3%), amoxicillin (9.2%), metronidazole (8.2%), cotrimoxazole (10%), ciprofloxacin (2.9%) and diphenhydramine (2.6%) (Table 2). The majority of medicines kept in households were prescribed by self (62%), health professionals (24.3%), and drug sellers (13.4%).

**Table 2:** Medicines present in households

**Total number of medicines present in homes (n = 821)**			
**Medicine category**	**Number (%)**	**Medicine category**	**Number (%)**
Analgesics	161 (19.6%)	Anti-diabetic drugs	3 (0.4%)
Antimalarials	128 (15.6%)	Antihypertensives	4 (0.5%)
Antibacterials	329 (40.1%)	Nutrient supplements	23 (2.8%)
Antihelmintics	9 (1.1%)	Corticosteroids	9 (1.1%)
Antifungals	14 (1.7%)	GIT drugs	36 (4.3%)
Antiretrovirals	34 (4.7%)	Antihistamines	26 (3.2%)
Others	45 (5.5%)		

GIT: Gastro-intestinal tract; n: sample size, %: percentage.

**Figure 1:**
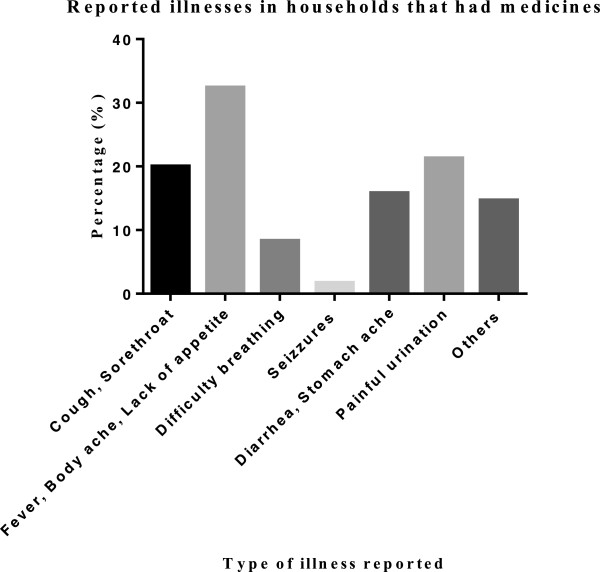
Disease symptoms in households that had medicines.

The study found that most of the medicines kept in households 55.7% (457/821) had clear labels on their package material. The average number of medicines kept in each household was 6 ± 5 with majority (68.1%) having between 1-to-10.Symptoms of malaria (fever, generalized body weakness), 34.1% and upper respiratory system infection (sore throat, cough, and dripping nose), 20.2% were most prevalent among household members in homes that kept medicines (Figure 1).

### Utilization and disposal of medicines kept in households

A high proportion 48.2% (396/821) of the medicines kept in households were for on-going treatment, a third was ‘leftover’ 30.5% while 21.6% were being kept for future use. Of the medicines used for on-going treatment in the households visited, 38.6% (153/396) were antibacterial agents. Antiretroviral 4.1%, antihypertensive 0.5% and anti-diabetic 0.4% medicines kept in homes were only for on-going treatment (Table 3).

**Table 3:** Use of the medicines present at home

**Category**	**Status of medicines at home (n = 821)**			
	**Current use**	**Leftover**	**Anticipated future use**	**Total (%)**
Analgesics	90 (10.9%)	42 (5.1%)	29 (3.5%)	161 (19.6%)
Antimalarials	42 (5.1%)	43 (5.2%)	43 (5.2%)	128 (15.6%)
Antibacterial	153 (18.6%)	122 (14.9%)	54 (6.6%)	329 (40.1%)
Antihelminth	1 (0.1%)	3 (0.4%)	5 (0.6%)	9 (1.2%)
Antifungals	6 (0.7%)	6 (0.7%)	2 (0.2%)	14 (1.7%)
Antiretrovirals	34 (4.1%)	-	-	34 (4.7%)
Nutrient supplements	13 (1.6%)	6 (0.7%)	4 (0.5%)	23 (2.8%)
GIT drugs	21 (2.6%)	8 (1.0%)	7 (0.9%)	36 (4.4%)
Antihypertensives	3 (0.4%)	-	1 (0.1%)	4 (0.5%)
Antidiabetic drugs	3 (0.4%)	-	-	3 (0.4%)
Corticosteroids	5 (0.6%)	2 (0.2%)	2 (0.2%)	9 (1.1%)
Antihistamines	12 (1.5%)	8 (1.0%)	6 (0.7%)	26 (3.2%)
Others	13 (1.6%)	20 (2.4%)	12 (1.5%)	45 (5.5%)
**Total**	**396 (48.2%)**	**260 (31.7%)**	**165 (20.1%)**	**821 (100%)**

%: Percentage, n: sample size.

Of the 313 respondents who reported presence of drugs in their households, 238 (76%) used these drugs without medical consultation. The ‘leftover’ medicines were disposed by giving to other sick members 33% (86/260), throwing away 10.8% (28/260), returning to the health facility 0.8% (2/260). The other ‘leftover’ medicines were kept for future use 55.4% (144/260).

### Multivariable logistic regression of the predictors of home storage of drugs in Northern Uganda

The model fitted with H-L statistic of 2.98 and probability (chi-square) of 0.56 on six (6) groups. The model yielded three significant predictors of home storage of medicines in Northern Uganda, past successful treatment (OR: 1.29, 95%CI: 0.95-1.77), regular income (OR: 1.76, 95%CI: 1.19-2.61) and female gender (OR: 0.63, 95%CI: 0.45-0.89).

## Discussion

Information from this study is of importance to the healthcare professionals and the community at large especially due to the risks that are associated with the presence of medicines in homes.

In the present study slightly over a third of all the households (35.1%) visited kept medicines. Patients are often prescribed medicines by health professionals for treatment of diagnosed illnesses of which a majority are used from home. In this regard, finding medicines in households in communities was not a surprise. However in the current study the respondents obtained and kept medicines in households for either emergency use or anticipated future treatment which is similar to findings of a study done in Sudan [2]. This could be an indicator of the challenges such as frequent drug stock outs, lack of adequate number of health professionals and inaccessibility of adequate healthcare facing the healthcare system in Northern Uganda which was affected by the two decades of armed conflict.

The prevalence of home storage of medicines found in this study is lower than that reported in studies done elsewhere in the world; 97.7% in a Sudanese study [2]; 94% in a study done in Iraq [5]. All these studies used similar methods of data collection although the current study had more than twice as large the sample size. Therefore this difference in the rates of home drug storage could have been due to the unique socio-economic factors in Northern Uganda. However this finding was closely similar to that of a study done in Northern United Arab Emirates which reported that 40% of all the households visited had drugs [3].

The medicines present in homes were mostly for on-going treatments which could indicate high prevalence of ill health in the community and were mostly used to treat symptoms of malaria and upper respiratory tract infections. With the need for quick recovery from ill health and the challenges of healthcare delivery in Northern Uganda, keeping drugs at home provides improved access for treatment especially in cases of emergencies. However with limited knowledge of proper drug storage, appropriate use and disposal in the communities, presence of medicines in households is likely to fuel irrational drug use due mainly to unintentional use among household members. This finding is comparable with reports from a previous study [9]. Inappropriate use of drugs may expose patients to adverse drug reactions, resistance development, financial loss and potentially prolonged illness [6].

The high prevalence of medicines in households of respondents who had attained a minimum of primary level of education and above (educated) was consistent with findings of a previous study [10]. This could be due to the increased health awareness among the more educated individuals and the need to take control of one’s health. Female respondents were more likely (OR: 0.63) to store drugs in their households and is comparable to the findings of a study done in Sudan [2]. This could be due to the central role women play in maintaining the health of family members especially children, a practice which is common in most parts of the world.

In this study, the most common medicines that were found in households included antibacterial (40.1%), analgesic (19.6%), and antimalarial (15.6%). The high prevalence of antibacterial agents in households could indicate wide spread use of these group of drugs in the communities and is consistent with the findings of a previous study [11]. However this is of public health concern as most of the respondents (76%) in households that had drugs reported using stored medicines without medical consultation in addition to sharing drugs among household members. This is consistent with the results from studies done elsewhere in the world, [3] (United Arab Emirates); [5] (Iraq) and [4] (Qatar). Sharing of medicines among individuals for whom the drugs were not intended could increase the risk of inappropriate drug use which potentially may exacerbate unwanted drug effects, treatment failure, morbidity and mortality [12-14]. Sharing of medicines among household members reflects the influence of social factors on the use of medicines in communities of Northern Uganda. The majority of households had antibacterial drugs such as co-trimoxazole (trimethoprim-sulfamethoxazole), metronidazole, ciprofloxacin and antimalarial (coartem). The use of these drugs is inherently associated with adverse drug reactions [15], which could be exacerbated by improper use commonly associated with self-medication. This is likely to increase the cost of treatment as patients have to spend on buying drugs in addition to treating the adverse effects which arise from their improper use [16].

Presence of medicines in households is a risk factor for encouraging inappropriate drug use such as using the antimicrobial drugs in illnesses when they are not indicated mainly due to the ease of access [17]. This inappropriate use of antimicrobials can potentially cause adverse drug reactions and resistance development [18]. Antimicrobial resistance is a worldwide problem with prevalence rates varying between different countries [19]. Resistance development to antimicrobial agents can occur even when these drugs are appropriately used however the progress is likely to be more rapid when they are used inappropriately [19]. With the high level of antimicrobial self-medication in Northern Uganda [20] the risk of resistance development to the common agents is a reality especially due to the inappropriate use of antibiotics which is common in self-medication.

Respondents who had prior successful treatment were more likely (OR: 1.29) to keep similar medications used in their households. This is mainly due to the confidence that patients acquire with time upon continued use of similar medications in addition to the ease of access of these drugs from the private sector. The major sources of medicines kept in homes were the private sector (drug shops, pharmacies and clinics) and ‘leftover’ drugs from previous prescriptions. Inadequate patient adherence to treatment in addition to poor prescription practices among health professionals could have contributed to the ‘leftover; drugs found in most of the households in communities of Northern Uganda [17,21]. The presence of ‘leftover’ drugs in households is a risk factor for self-medication [1] and the associated effects.

The medicines in homes are kept in unregulated conditions of temperature and humidity which could accelerate drug degradation and expiry [22]. However due to inability to assess the expiry of drugs and the effect of storage conditions on the potency of medicines in households it is possible that these drugs could be kept and used beyond their expiry dates [3]. This became more apparent with the presence of self-initiated use of drugs kept at home which exposes patients to risks such as adverse drug reactions, accidental poisoning and resistance development [6]. In the present study it was difficult to assess the expiry dates of the drugs kept in homes as most of the medicines were in secondary packages.

In this study, there was a very low rate (0.2%) of reported return of ‘leftover’ or unwanted medicines in households to healthcare facilities for proper disposal and is similar to what was observed in other studies [4]. This is surprising as Uganda enacted the National drug policy in 2002 which clearly spells out methods of proper drug disposal and this should be of concern to the policy makers. However this could be due to the reluctance among health professionals in providing patients with information on how to properly handle or use medicines in households [23]. The improper drug disposal methods such as giving out the ‘unwanted’ or ‘left over’ drugs to other sick members or throwing a way to the common rubbish pits as found in the current study could endanger the environment in addition to promoting irrational drug use in the community [3,24].

Solid dosage formulations (tablets and capsules) were the major forms of medicines kept in households in Northern Uganda. This could be due to their ease of administration and acceptability in the community [2]. The average number of medicines kept in each household was six (6 ± 5) with most homes keeping between 1–10 medicines. This was similar to the mean number of medicines found in homes in studies done elsewhere, Qatar 6.0 [4], Sudan 4.4 [2] and Saudi Arabia 8.0 [25]. This finding points to the presence of inappropriate prescription practice among health professionals in addition to poor treatment adherence among the respondents [3].

Respondents who reported having regular income were about twice more likely (OR: 1.76) to keep medicines in their households. This is contrary to a study done in Qatar [4] and could be due to the difference in the data collection methods as this study used telephone calls as opposed to face-to-face interview used in our study. The differences in healthcare infrastructural development between Northern Uganda and Qatar could have also contributed to the difference in the findings of these studies. The challenges of healthcare delivery such as frequent drug stock outs, and lack of medical personnel common in Northern Uganda [5,26], potentially influence communities to seek alternative ways to access treatment including storage of medicines in homes as standby drugs [27]. The ease of access of medicines from the private sector in the community due mainly to inadequate regulation in addition to availability of money among respondents potentially influences home storage of medicines in Northern Uganda.

Provision of awareness to the communities on the risks of home drug storage and self-medication through health campaigns in addition to drug regulation may be a better approach than focusing only on drug regulation in tackling the challenge of home drug storage and utilization in Northern Uganda.

Interventions targeted on health professionals in the communities of Northern Uganda such as trainings on good prescription practices and dispensing of drugs would help in reducing the risk of household drug storage. In addition incorporation of community education on treatment compliance, risks of home medicine storage and their use without medical consultation and proper disposal of leftover drugs in patient care will go a long way in solving the problem of home drug storage in Northern Uganda and the associated risks.

## Conclusion

Medicines are kept in over a third of the households in communities of Northern Uganda with antibacterial drugs being the most common and are often used without medical consultation. Majority of household members lack knowledge on proper disposal of drugs which remain from previous treatment. Past successful treatment, regular income and female gender predict home storage of drugs in communities of Northern Uganda.

## Competing interests

The authors of this article do not have any conflict of interest to declare.

## Authors' contributions

MO, JO and CO participated in the design, coordination analysis and writing up of the manuscript, MO made the first draft of the manuscript. GSB and PW participated in the review of the manuscript. All authors read and approved the final manuscript.

## Authors’ information

1^*^ PhD Pharmacology fellow Makerere University College of Health Sciences. GSB is a Lecturer in the Department of Pharmacology and Therapeutics Makerere University College of Health Sciences. PW, JO, and CO are Professors of Pharmacology in the Department of Pharmacology and Therapeutics Makerere University College of Health Sciences.

## Pre-publication history

The pre-publication history for this paper can be accessed here:

http://www.biomedcentral.com/1471-2458/14/650/prepub
